# *Curvicladiellapaphiopedili* sp. nov. (Hypocreales, Nectriaceae), a new species of orchid (*Paphiopedilum* sp.) from Guizhou, China

**DOI:** 10.3897/BDJ.10.e80122

**Published:** 2022-04-05

**Authors:** Lian-chai Song, Lu Huang, Ling-ling Liu, Yao Feng, Li-li Wang, Zuo-yi Liu

**Affiliations:** 1 College of Agriculture, Guizhou University, Guiyang, China College of Agriculture, Guizhou University Guiyang China; 2 Scientific research administration office, Guizhou Academy of Agricultural Sciences, Guiyang, China Scientific research administration office, Guizhou Academy of Agricultural Sciences Guiyang China; 3 Institute of Plant Protection, Guizhou Academy of Agricultural Sciences, Guiyang, China Institute of Plant Protection, Guizhou Academy of Agricultural Sciences Guiyang China; 4 Guizhou Institute of Soil and Fertilizer, Guizhou Academy of Agricultural Sciences, Guiyang, China Guizhou Institute of Soil and Fertilizer, Guizhou Academy of Agricultural Sciences Guiyang China; 5 School of Ethnic Medicine, Guizhou Minzu University, Guiyang, China School of Ethnic Medicine, Guizhou Minzu University Guiyang China; 6 Guizhou Key Laboratory of Agricultural Biotechnology, Guizhou Academy of Agricultural Sciences, Guiyang, China Guizhou Key Laboratory of Agricultural Biotechnology, Guizhou Academy of Agricultural Sciences Guiyang China

**Keywords:** *Curvicladiella*, morphology, phylogeny, *Paphiopedilum*, taxonomy

## Abstract

**Background:**

An asexual fungus, collected from diseased leaves of *Paphiopedilum* sp. from Guizhou Province, China, and based on the phylogenetic analyses and morphological characters, it was identified as a new species in *Curvicladiella*. The genus *Curvicladiella* are recorded for the first time for China.

**New information:**

The morphology of *Curvicladiellapaphiopedili* sp. nov. is characterised by penicillate conidiophores with a stipe, dull, tapering towards the apex, the curved stipe extension and cylindrical conidia. In the phylogenetic analyses of combined cmdA, his3, ITS, LSU, tef1 and tub2 sequence data, this taxon was clustered as sister to *Curvicladiellacignea* within Nectriaceae.

## Introduction

Nectriaceae (order Hypocreales) includes many important plant and human pathogens and some species have been used as biodegrading and biocontrol agents in industrial and commercial applications (Lombard et al. 2015). Based on molecular studies, many sexual genera in Nectriaceae were placed in *Nectria* sensu lato (Rehner and Samuels 1995, Rossman et al. 1999). However, *Nectria* sensu stricto is restricted to the type species *N.cinnabarina* (Tode) Fr et al. with tubercularia-like asexual morphs (Rossman 2000, Hirooka et al. 2012). A number of studies have treated taxonomic concepts within Nectriaceae, based on multi-gene phylogenetic inference (Lombard et al. 2010a, Lombard et al. 2010b, Chaverri et al. 2011, Gräfenhan et al. 2011, Schroers et al. 2011, Hirooka et al. 2012,Lombard and Crous 2012a, Lombard and Crous 2012b, Lombard et al. 2014a, Lombard et al. 2014b). Lombard et al. (2015) provided a phylogenetic backbone tree for Nectriaceae, based on combined sequence data of 10 gene regions. *Curvicladiella* is one of the genera in the Nectriaceae.

Decock and Crous (1998) established *Curvicladium* (as *Curvicladiella*) with *C.cigneum* (as *Curvicladiellacignea*) as the type species. The genus is distinct from morphologically-similar genera, such as *Cylindrocladium* Morgan, *Cylindrocladiella* Boesew, *Gliocladiopsis* Saksena, *Falcocladium* Silveira, Alfenas, Crous, Wingf and *Xenocylindrocladium* Decock, Hennebert, Crous by having cylindrical conidia and stipe extensions (Decock and Crous 1998). *Curvicladiellacignea* is the only species in the genus.

Based on the phylogenetic analyses and morphological characters, the fungus collected from diseased leaves of *Paphiopedilum* sp. is identified as a new species in *Curvicladiella*, the artificial infection test shows that it is a pathogen and the specific infection process has been described by Song et al. (Song et al. 2020). *Paphiopedilum* is known as“slipper orchids”, has a high ornamental value and can be used as household bonsai and garden plants(Luan et al. 2019).

## Materials and methods

### Sample collection and isolation

Diseased orchid leaves were collected from Guizhou Botanical Garden, Guizhou Province, China (in August 2019). The samples were brought to the laboratory in envelopes, photographed and identified. Pieces of leaves (5 × 5 mm), half of which were diseased and half healthy, were sterilised by 75% ethanol for 5–10 s, rinsed three times with sterilised distilled water, placed on potato dextrose agar (PDA) and incubated at 25°C for two days (Fang 2001). Mycelia were transferred to PDA and incubated for ten days at 25°C to obtain the pure cultures. The morphological characters of the fungi obtained from the diseased leaves collected in the field and cultured with PDA, and the fungi obtained from the diseased leaves after an artificial infection test were observed using a Nikon SMZ 745 stereomicroscope. Measurements were made using Image Frame Work.

Pure cultures were deposited in Guizhou Culture Collection (GZCC) Guizhou, China and Mae Fah Luang University Culture Collection (MFLUCC), Chiang Rai, Thailand. Herbarium specimens were deposited in the Guizhou Academy of Agricultural Sciences (GZAAS), Guiyang, China and the Herbarium of Mae Fah Luang University (MFLU), Chiang Rai, Thailand.

### DNA extraction, PCR amplification and sequencing

The fungal mycelia were scraped from the pure culture growing on PDA for ten days at 25ºC. DNA was extracted using the Ezup Column Fungi Genomic DNA Purification Kit (Sangon Biotech, China). Six gene regions, the 28S large subunit rDNA (LSU), calmodulin (cmdA), histone H3 (his3), internal transcribed spacer region and intervening 5.8S nrRNA gene (ITS), translation elongation factor 1-alpha (tef1) and β-tubulin (tub2) gene were amplified by the primer pairs LR0R and LR5(Vilgalys and Hester 1990, Rehner and Samuels 1994), CAL-228F and CAL2Rd (Carbone and Kohn 1999, Groenewald et al. 2013), CYLH3F and CYLH3R (Crous et al. 2004), ITS5 and ITS4 (White et al. 1990), EF1-728F and EF2 (O'Donnell et al. 1998, Carbone and Kohn 1999), T1 and CYLTUB1R (O'Donnell and Cigelnik 1997, Crous et al. 2004), respectively. Polymerase chain reaction (PCR) was carried out in 25 µl reaction volume containing 12.5 µl 2 × PCR Master Mix (Sangon Biotech, China), 9.5 µl ddH_2_O, 1µl of each primer and 1µl DNA template. The PCR products were examined by using 1.2% agarose electrophoresis gel, stained with ethidium bromide and were purified and sequenced by Sangon Biotech (Shanghai) Co. Ltd, China. The nucleotide sequences were submitted in GenBank.

### Phylogenetic analyses

Phylogenetic analyses were performed using combined sequence data with six gene regions, LSU, cmdA, his3, ITS, tef1 and tub2. Related strains of *Curvicladiella* (Table 1) were referred to Lombard et al. (2015). Sequences were obtained from GenBank. The sequences were aligned using the online multiple alignment programme MAFFT v.7 (http://mafft.cbrc.jp/alignment/server) (Standley 2013). The alignments were checked visually and optimised manually by using BioEdit v. 7.2.6.1.

Maximum Likelihood (ML) analysis was performed using RaxmlGUI 1.3.1 (Silvestro and Michalak 2012). The optimal RAxML tree search was conducted with 1000 bootstrap replicates and the default algorithm was used from a random starting tree for each replicate. The final tree was selected from amongst suboptimal trees from each replicate by comparing likelihood scores under the GTR+GAMMA substitution model.

Bayesian analyses were carried out using MrBayes 3.2 (Huelsenbeck 2012). MrModeltest 2.2 was used to choose the best-fit evolutionary model (Nylander 2004). Posterior probabilities (PP) (Rannala and Yang 1996, Zhaxybayeva and Gogarten 2002) were determined by Markov Chain Monte Carlo sampling (MCMC) in MrBayes v. 3.2. Six simultaneous Markov chains were run for 10 million generations and trees were sampled every 1000^th^ generation. The temperature values were lowered to 0.15, burn-in was set to 0.25 and the run was automatically stopped as soon as the average standard deviation of split frequencies reached below 0.01.

The resulting trees of Maximum Likelihood and Bayesian were visualised with Fig Tree v.1.4.0. The layouts were undertaken using Microsoft Powerpoint 2010 and Adobe Photoshop CS6.

## Taxon treatments

### Curvicladiella paphiopedili

Lian-Chai Song，Jing Yang, Zuo-Yi Liu, 2019 sp. nov.

47DDD2AC-EB68-5625-AE33-768FDF9CDA7E

http://www.indexfungorum.org/names:IF558310

Facesoffungi number:FOF 09697

#### Materials

- **Type status:** Holotype. **Occurrence:** recordNumber: zwy-dl4-2; recordedBy: Lian Chai Song; **Taxon:** scientificName: *Curvicladiellapaphiopedili*; class: Sordariomycetes; order: Hypocreales; family: Nectriaceae; genus: Curvicladiella; **Location:** locationRemarks: China, Gui Zhou Province, Guiyang City, Guizhou Botanical Garden, 26°37'N, 106°43'E, 13 August 2019; **Event:** habitat: Terrestrial; fieldNotes: diseased leaves of *Paphiopedilum* sp.; **Record Level:** type: Stilllmage; language: English; collectionID: MFLU 20-0203
- **Type status:** Isotype. **Occurrence:** recordNumber: zwy-dl4-2; recordedBy: Lian Chai Song; **Taxon:** scientificName: *Curvicladiellapaphiopedili*; class: Sordariomycetes; order: Hypocreales; family: Nectriaceae; genus: Curvicladiella; **Location:** locationRemarks: China, Gui Zhou Province, Guiyang City, Guizhou Botanical Garden, 26°37'N, 106°43'E, 13 August 2019; **Event:** habitat: Terrestrial; fieldNotes: diseased leaves of *Paphiopedilum* sp.; **Record Level:** type: Stilllmage; language: English; collectionID: GZAAS 19-2061

#### Description

**Figure 1.**
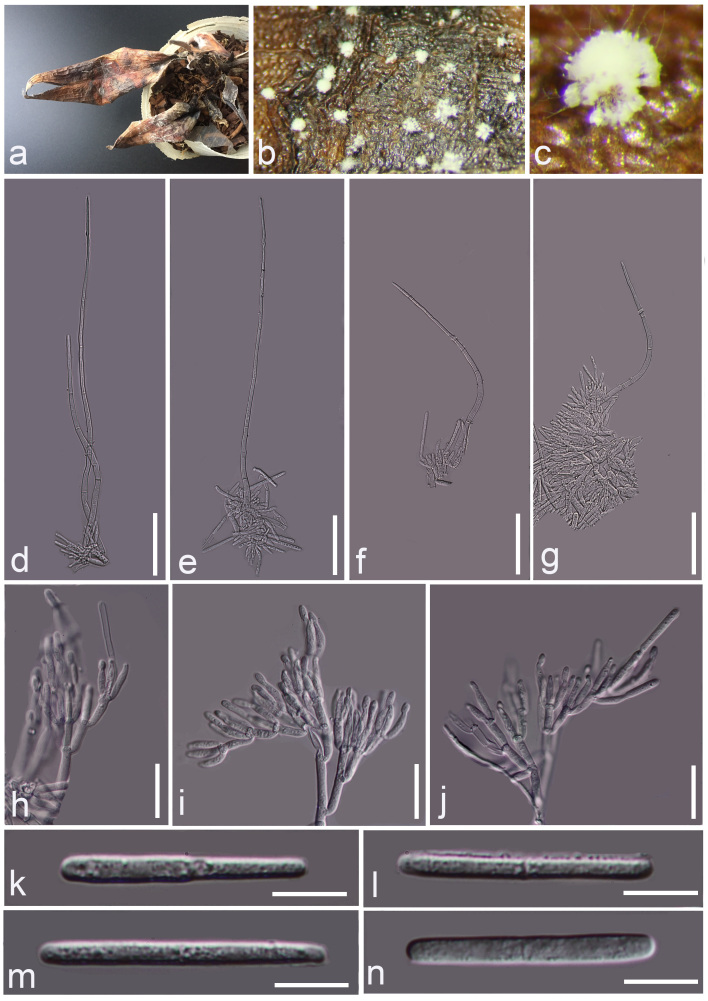
*Curvicladiellapaphiopedili*. **a** The diseased leaves were withered; **b, c** Conidiomata; **d–g** Stipes extension and conidiogenous cells; **h–j** Conidiogenous cells and conidiophores; **k–n** Conidia. Scale bars: **d–g**=50 µm, **h–j**=20 µm, **k–n**=10 µm.

The characters of pathogenic fungi on the leaves were identified through an artificial infection test. **Asexual morph**: Conidiomata white, scattered, hairy. Conidiophores straight to flexuous, consisting of a stipe bearing a penicillate arrangement of fertile branches, stipe septate, hyaline, smooth; stipe extensions septate, straight or curved, dull and tapering towards the apex, 128.5–549.9 µm long, (x̄= 288.1 µm, n = 20). The primary branches of conidiogenous apparatus aseptate, 9.3–17.5 × 2.6–3.7 μm; secondary branches aseptate, 9.9–19.1 × 2.5–3.9 μm; tertiary branches aseptate, 9.5–17.6 × 2.6–3.7 μm; quaternary and additional branches (–6) aseptate, 11–16.3 × 2.5–3.9 μm, each terminal branch producing 2–4 phialides; phialides doliiform to reniform, hyaline, aseptate, apex with minute periclinal thickening and inconspicuous collarette. Conidia cylindrical, rounded at both ends, straight, 1-septate, hyaline, (30.5–) 31.2–37.2 (–42.0) × (2.6–) 2.9–3.5 (–3.9) µm, (x̄= 34.2 × 3.2 µm, n = 20) (Fig. 1). **Sexual morph**: not observed.

**Figure 2.**
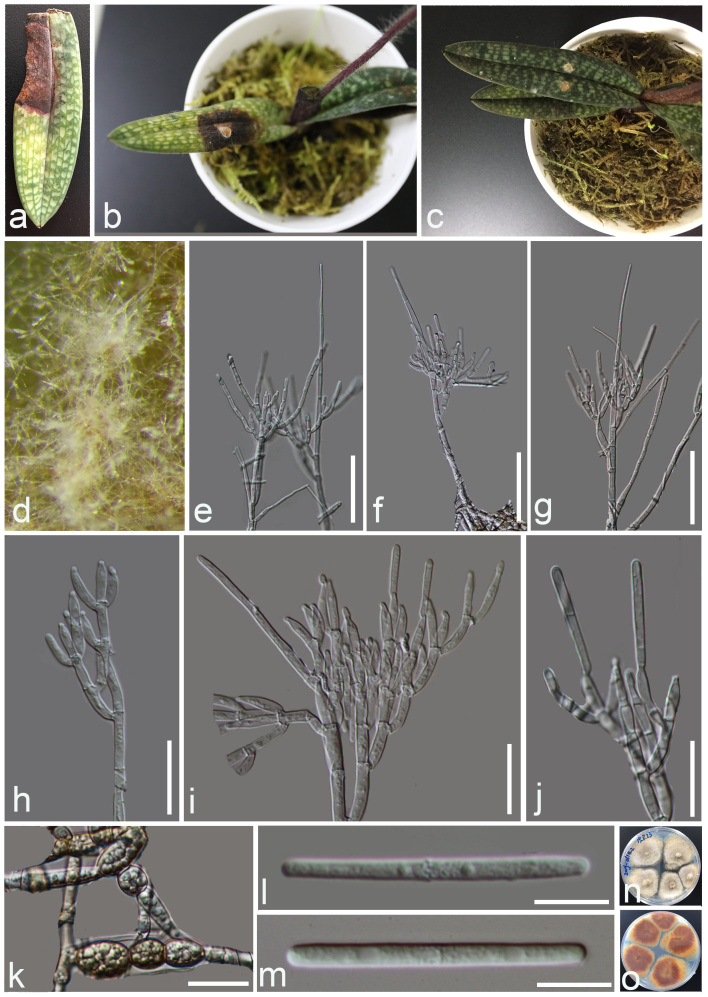
*Curvicladiellapaphiopedili*. (MFLU 20-0203, holotype) **a** *Paphiopedilum* diseased leaf in the field; **b** The healthy leaves diseased after inoculating the mycelial PDA plug of *Curvicladiellapaphiopedili* ; **c** The healthy leaves did not become infected after being inoculated with free PDA plug as control; **d** Colonies on PDA producing conidia masses; **e–j** Conidiophores, conidiogenous cells and stipes extension; **k** Chlamydosporae; **l, m** Conidia; **n, o** Culture on PDA; (**n**) from above, (**o**) from below. Scale bars: **e–g**=50 µm, **h–k**=20 µm, **l, m**=10 µm.

The characters of fungus obtained from the diseased leaves collected in the field that were cultured with PDA: after 10 days at 25°C on PDA, colonies reached 47 mm in diam. Beige to pale yellow colony on the surface, brown in reverse with irregular margins, extensive sporulation on the medium surface. Conidiophores straight to flexuous, consisting of a stipe bearing a penicillate arrangement of fertile branches, stipe extensions septate, straight or slightly flexuous, 104.4–153.0 µm long, (x̄= 128.7 µm, n = 10). The primary branches of conidiogenous apparatus aseptate, 8.9–17.8 × 2.7–3.4 μm; secondary branches aseptate, 7.8–14.0 × 2.5–5.9 μm; tertiary branches aseptate, 8.9–17.7 × 2.3–3.5 μm; quaternary and additional branches (–6) aseptate, 9.3–16.7 × 2.3–3.7 μm, each terminal branch producing 2–4 phialides; phialides doliiform to reniform, hyaline, aseptate, apex with minute periclinal thickening and inconspicuous collarette. Conidia cylindrical, rounded at both ends, straight, 1-septate, hyaline, (38.5–) 45.2–56.6 (–63.2) × (2.2–) 2.9–4.2 (–4.9) µm, (x̄= 50.9 × 3.5 µm, n = 40). Chlamydospores thick-walled, ellipsoidal or sphaeropedunculate, brown to hyaline, (9.0–) 11.9–20.7 (–23.1) × (8.1–) 8.9–12.8 (–15.4) µm, (x̄= 16.3 × 10.8 µm, n = 20) (Fig. 2).

Material: ex-type living culture, MFLUCC 20-0110.

#### Etymology

Refers to the genus name *Paphiopedilum*.

## Analysis

**Phylogenetic analyses**

**Figure 3.**
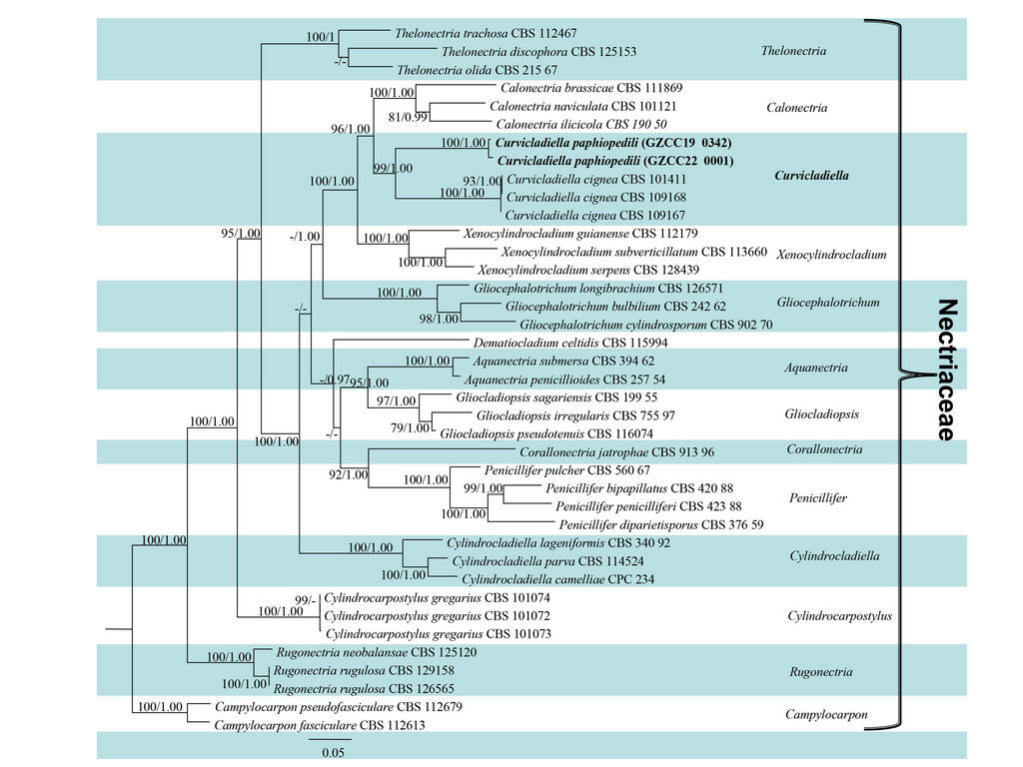
The RAxML tree, based on analysis of cmdA, his3, ITS, LSU, tef1 and tub2 sequences data. Bootstrap support values for ML and Bayesian greater than 75% and 0.95 were given near nodes, respectively. The tree was rooted with *Campylocarponfasciculare* and *Campylocarponpseudofasciculare*. The new isolate are shown in bold.

The final alignment consists of the new species and the fungus obtained from the diseased leaves after use of the new species to infect the heathy Paphiopedilum and other genera of the families Nectriaceae. Additionally, the alignment of combined cmdA, his3, ITS, LSU, tef1 and tub2 sequence data comprised a total of 3877 characters with gaps (734bp for cmdA, 529bp for his3, 616bp for ITS, 840bp for LSU, 548bp for tef1 and 610bp for tub2). The dataset comprised 39 taxa with *Campylocarponfasciculare* and *C.pseudofasciculare* as the outgroup taxa. The best scoring RAxML tree is shown in Fig. 3, with the Bayesian tree (not shown) having a similar topology with the ML tree. *Curvicladiellapaphiopedili* was clustered as sister taxon to *C.cignea* within Nectriaceae with high support (99/1.00) (Fig. 3).

## Discussion

Morphologically, *Curvicladiellapaphiopedili* is similar to species in *Calonectria*, *Cylindrocladium* and *Xenocylindrocladium*, but distinct in having ellipsoidal or sphaeropedunculate chlamydospores (Fig. 2k), dull, tapering towards the apex (Fig. 1d and e, Fig. 2e–g) and curved extension stipes (Fig. 1f and g), without obpyriform, ovoid, ellipsoidal or sphaeropedunculate vesicles (Lombard et al. 2010a, Pham et al. 2019) or coiled stipes (Decock et al. 1997). The morphology of *Curvicladiellapaphiopedili* is different from the type species *Curvicladiellacignea* in the size of stipe extensions and conidia, without the swollen cell below the apical septum; on the other hand, the stipe extensions of *Curvicladiellacignea* is curved obviously, while *Curvicladiellapaphiopedili* is not. The stipe extensions of *Curvicladiellapaphiopedili* are 128.5–549.9 µm long, the *Curvicladiellacignea* is 110-200 µm long. The conidia of *Curvicladiellapaphiopedili* are (38.5–) 45.2–56.6 (–63.2) × (2.2–) 2.9–4.2 (–4.9) µm, the *Curvicladiellacignea* are (28–) 33–36 (–38) × 2.5–3µm. In the phylogenetic analyses, the two taxa of *Curvicladiella* formed a well-supported monoclade and *Curvicladiellaaphiopedili* represented a distinct lineage (Fig. 3).

## Supplementary Material

XML Treatment for Curvicladiella paphiopedili

## Figures and Tables

**Table 1.** Taxa or selected taxa used in this study and their GenBank accession numbers. The type species have T as superscript and the newly-generated sequences have been highlighted in bold.

Taxa	Isolate numbers	GenBank Accession numbers					
		LSU	CMDA	HIS3	ITS	TEF1	TUB2
*Aquanectriapenicillioides*	CBS 257.54	KM231613	KM231275	–	KM231743	KM231865	KM232000
*Aquanectriasubmersa*	CBS 394.62 ^T^	KM231612	–	KM231458	HQ897796	–	KM231999
*Calonectriabrassicae*	CBS 111869	GQ280698	GQ267382	DQ190720	GQ280576	FJ918567	AF232857
*Calonectriailicicola*	CBS 190.50 ^T^	GQ280727	AY725764	AY725676	GQ280605	AY725726	AY725631
*Calonectrianaviculata*	CBS 101121 ^T^	GQ280722	GQ267399	GQ267252	GQ280600	GQ267317	GQ267211
*Campylocarponfasciculare*	CBS 112613 ^T^	HM364313	KM231297	JF735502	AY677301	JF735691	AY677221
*Campylocarponpseudofasciculare*	CBS 112679 ^T^	HM364314	KM231298	JF735503	AY677306	JF735692	AY677214
*Corallonectriajatrophae*	CBS 913.96 ^T^	KM231611	KM231273	KM231457	KC479758	KM231863	KC479787
*Curvicladiellacignea*	CBS 101411	JQ666075	KM231285	KM231459	KM231744	KM231866	KM232001
*Curvicladiellacignea*	CBS 109168	JQ666074	KM231286	KM231460	KM231745	KM231868	KM232003
*Curvicladiellacignea*	CBS 109167 ^T^	AY793431	KM231287	KM231461	AF220973	KM231867	KM232002
***Curvicladiellapaphiopedili***	**MFLUCC 20-0110 ^T^**	**MT279199**	**MT294104**	**MT294105**	**MT279198**	**MT294103**	**MT294102**
***Curvicladiellapaphiopedili***	**GZCC22-0001**	**OM899803**	–	–	**OM903885**	–	–
*Cylindrocarpostylusgregarius*	CBS 101074	KM231614	KM231291	–	KM231746	KM231869	KM232004
*Cylindrocarpostylusgregarius*	CBS 101072 ^T^	JQ666084	KM231292	–	KM231747	KM231870	KM232005
*Cylindrocarpostylusgregarius*	CBS 101073	JQ666083	KM231293	KM231465	KM231748	KM231871	KM232006
*Cylindrocladiellacamelliae*	CPC 234 ^T^	JN099249	KM231280	AY793509	AF220952	JN099087	AY793471
*Cylindrocladiellalageniformis*	CBS 340.92 ^T^	JN099165	KM231279	AY793520	AF220959	JN099003	AY793481
*Cylindrocladiellaparva*	CBS 114524 ^T^	JN099171	KM231281	AY793526	AF220964	JN099009	AY793486
*Dematiocladiumceltidis*	CBS 115994 ^T^	AY793438	KM231274	–	AY793430	KM231864	–
*Gliocephalotrichumbulbilium*	CBS 242.62 ^T^	AY489732	KM231283	KF513326	–	KM231892	DQ377831
*Gliocephalotrichumcylindrosporum*	CBS 902.70T	JQ666077	KM231284	KF513353	DQ366705	KF513408	DQ377841
*Gliocephalotrichumlongibrachium*	CBS 126571 ^T^	KM231686	KM231282	KF513367	DQ278422	KF513435	DQ377835
*Gliocladiopsisirregularis*	CBS 755.97 ^T^	JQ666082	KM231278	JQ666023	AF220977	KF513449	JQ666133
*Gliocladiopsispseudotenuis*	CBS 116074 ^T^	JQ666080	KM231277	JQ666030	AF220981	JQ666099	JQ666140
*Gliocladiopsissagariensis*	CBS 199.55 ^T^	JQ666078	KM231276	JQ666031	JQ666063	JQ666106	JQ666141
*Penicilliferbipapillatus*	CBS 420.88 ^T^	KM231608	KM231270	KM231454	KM231740	KM231860	KM231996
*Penicilliferdiparietisporus*	CBS 376.59 ^T^	KM231609	KM231271	KM231455	KM231741	KM231861	KM231997
*Penicilliferpenicilliferi*	CBS 423.88 ^T^	KM231607	KM231269	KM231453	KM231739	KM231859	KM231995
*Penicilliferpulcher*	CBS 560.67 ^T^	KM231610	KM231272	KM231456	KM231742	KM231862	KM231998
*Rugonectrianeobalansae*	CBS 125120	HM364322	KM231294	KM231466	KM231750	KM231874	HM352869
*Rugonectriarugulosa*	CBS 129158	JF832761	KM231295	KM231467	JF832661	KM231872	JF832911
*Rugonectriarugulosa*	CBS 126565	KM231615	KM231296	KM231468	KM231749	KM231873	KM232007
*Thelonectriadiscophora*	CBS 125153	HM364307	KM231327	KM231489	HM364294	KM231897	HM352860
*Thelonectriaolida*	CBS 215.67 ^T^	HM364317	KM231325	KM231487	AY677293	HM364345	KM232024
*Thelonectriatrachosa*	CBS 112467 ^T^	HM364312	KM231326	KM231488	AY677297	KM231896	AY677258
*Xenocylindrocladiumguianense*	CBS 112179 ^T^	JQ666073	KM231289	KM231463	AF317348	KM231895	AF320197
*Xenocylindrocladiumserpens*	CBS 128439 ^T^	KM231688	KM231290	KM231464	AF220982	KM231894	AF320196
*Xenocylindrocladiumsubverticillatum*	CBS 113660 ^T^	KM231687	KM231288	KM231462	AF317347	KM231893	AF320196
T Ex-type and ex-epitype cultures. CBS: CBS-KNAW Fungal Biodiversity Centre, Utrecht, The Netherlands; CPC: P.W. Crous collection.							
